# Recovery of carotenoids as bioactive compounds from peach pomace by an eco-friendly ultrasound-assisted enzymatic extraction

**DOI:** 10.1007/s13197-024-06001-4

**Published:** 2024-05-18

**Authors:** Kübra Nur Han, Hilal Meral, Aslıhan Demirdöven

**Affiliations:** Faculty of Engineering and Architecture, Food Engineering Departement, Tokat Gaziosmanpasa University, 60150 Tokat, Turkey

**Keywords:** Ultrasound, Enzyme, Extraction, Peach processing waste

## Abstract

The industrial processing of fruits generates by-products. These by-products serve as a source of valuable bioactive compounds. In this study, carotenoid was extracted from peach pomace (PP) by using the ultrasound-assisted enzymatic extraction (UAEE), an eco-friendly method. The process conditions ensuring the highest carotenoid content and b* color value for UAEE were detected by response surface methodology (RSM). To demonstrate the effectiveness of the ultrasonic process, enzymatic extraction was carried out at the optimum point. Physicochemical (pH, titratable acidity, total soluble solids), color (L*, a*, b*, chroma value (ΔC) and color difference (ΔE), total phenolic compound (TPC) and antioxidant activity analyses (ABTS and FRAP) were carried. When the analysis results evaluated, the highest b* color parameter, TPC (761.10 mg gallic acid/L), ABTS (1933.33 mg Trolox/L) and FRAP (52.66 µmol Trolox/L) results of the extracts was observed with UAEE method. The study shows that ultrasound based upon the cavitation event was increased efficiency of enzymatic reaction with higher extraction yield and this provided in higher amounts of carotenoid and bioactive compounds. In other respects, when obtained carotenoid extracts are used in food formulations compatible with their acidic structure, they will contribute to protection of the product and minimizing color losses.

## Introduction

The industrial production of fruit and vegetable juices constitutes nearly 50% waste including cores, peels, pomace, unripe and/or damaged fruits and vegetables. These by-products are used as renewable resources because of their low-cost and abundant availability to improve new functional products, natural ingredients, and generate value-added products for reducing negative environmental impacts. In addition to preventing environmental pollution by evaluating industrial wastes and transforming them into high value-added products, advantages such as reducing external dependency, generating commercial income, obtaining functional coloring agents, and creating alternatives for prebiotic products with dietary fiber production can be achieved (Pimentel-Moral et al. 2020; Vorobyova and Skiba 2021).

As the number of fruit processing factories increases day by day, the amount of waste generated is also increasing. In this sense, peach is one of the fruits that produce a large amount of waste as a result of processing, and it is used frequently in food industry. By-products of fruit processing, especially during cutting and pressing, mainly consist of peel, seed and pomace. Peach pomace (PP), which consists of fruit flesh and peel, constitutes is the largest portion of peach processing wastes (Plazzotta et al. 2020). PP include high amounts of bioactive compounds and biopolymers including polyphenols, vitamins and/or natural colorants (Sagar et al. 2018). PP is an important source of carotenoids, one of these bioactive compounds. The content of carotenoids responsible for the yellow-orange color of PP varies between 1.1 and 22.3 mg/100 g, and 26–48% of this amount is β-carotene, 18–34% *Z*-γ-carotene and 10–27% of lycopene as most dominant groups (Ordóñez-Santos et al. 2015).

Carotenoids are the most important bioactive compounds which have lipophilic character in nature. These compounds which have an isoprene skeleton, are composed of 40 carbon atoms are responsible for the orange, red or yellow color in a variety of fruits and vegetables (Ambati et al. 2019). These are the most important photosynthetic pigments ensuring the protection of chlorophyll membrane from photo oxidative losses. Carotenoids have wide application areas in the food industry as a natural colorant. Also, they can be used in product development because of their potential antioxidant activities (Fernández-López et al. 2020).

Different techniques including conventional extraction processes like maceration and/or Soxhlet extraction and novel extraction processes like pulse electric field, ultrasound, supercritical fluid extraction, microwave and hydrodynamic cavitation are used to extract bioactive compounds from PP (Keisandokht et al. 2018). The conventional extraction methods have many disadvantages. For example, high amount of solvent utilization, longer extraction time and low extraction yield. Recently, lots of studies in literature have concentrated on new extraction techniques to overcome these drawbacks (Dastan et al. 2022). One of these new techniques, ultrasound assisted extraction (UAE) is an inexpensive and simple in comparison with conventional extraction methods. Ultrasound is identified as sound waves with frequencies above the threshold for human hearing (> 16 kHz). These sound waves cause an increase in pressure and temperature. The ultrasound effect is explained by the shock waves that occur as a result of the cavitation caused by these pressure and temperature changes. In food industry, ultrasound devices operating in frequencies from 20 kHz to 10 MHz are used. The extraction of lipids, proteins, carotenoids, hemicelluloses, and aromatic compounds with UAE are reported in the literature (Demirdoven et al. 2021).

In recent studies, the use of enzymes in UAE has become widespread in order to perform the extraction process more effectively and quickly. Enzymes are widely utilized in fruit juice industry to break down cell walls of plant and tissues for progress in juice yield. Therefore, extraction of bioactive compounds becomes easier due to the increase in cell permeability during juice processing. However, the use of enzymes alone in the bioactive compound extraction increases the processing time and thus increases the processing cost. Studies in recent years have shown that the use of ultrasound waves during enzymatic extraction has positive results on the process (Liu et al. 2014; Tchabo et al. 2015; Kaur et al. 2022). For this reason, different new methods including UAEE which is combinational usage of enzyme and UAE have been improved to shorten the processing time by increasing the extraction yield and enhancing the quality of the extracts by providing effective extraction conditions (Baysal and Demirdoven 2012; Tan et al. 2020; Xue et al. 2021; Gao et al. 2022).

In last years, a lot of research have focused on the content of polyphenolic compounds in fruit and vegetable processing by-products, the production of bioactive compounds from these products (Zengin et al. 2020). Carotenoid extracts exemplify one of the most important recovered phytochemical compounds. There are many studies in the literature on the extraction of carotenoids from juice processing by-products (Sharma et al. 2021). But there is not study has been presented in which carotenoid extracts produced from PP including the optimization of UAEE conditions as green technology. Therefore, the aim of this study is that optimization of carotenoid extraction conditions to enable maximum achievement of carotenoids and production as color pigment with enzymatic and UAE using nature-friendly methods from PP, which is a food industrial waste.

## Materials and methods

### Materials

The PP used in the study was provided by Dimes Food Ind. and Trade. Inc. (Tokat-TURKEY) and stored at − 18 °C in the laboratories of Tokat Gaziosmanpasa University Food Engineering Department (Tokat-TURKEY) until the extraction stage. Before extraction, the samples were thawed at + 4 °C. Pectinex Ultra Color (Novozymes-Denmark) enzyme preparate was used for enzymatic extraction. The enzyme preparate having pectin lyase and polygalacturanase activity (7700 PECTU/ml) was stored at + 4 °C until the extraction. The pomace was corresponding to 74.4%, the peel was 19.8%, the stone and other parts were 5.8% of the fruit weight. The pH value of PP was 4.06 ± 0.02 and titratable acidity was 1.07 ± 0.07% in terms of malic acid. The brix of PP was determined 11.0 ± 0.1% and it had 85.2% moisture content. The color values for PP were L*(brightness) = 49.28 ± 0.12, a*(redness) = 8.92 ± 0.16, b*(yellowness) = 37.37 ± 0.69.

### Chemicals and reagents

Folin–Ciocalteau reagent (Cas No. 12111-13-6, Germany, Merck); 2,2-diphenyl-1-picrylhydrazyl (DPPH) (Cas No. 1898-66-4, Steinheim, Germany); 2,2-Azino-bis-(3-ethylbenzothiazoline-6-sulfonic acid) diammonium salt (ABTS) (Cas No. 30931-67-0, Steinheim, Germany); 2,4,6-Tripyridyl-*s*-triazine (TPTZ) (Cas No. 3682-35-7, Steinheim, Germany); ± -6-hydroxy-2,5,7,8-tetramethylchroman-2-carboxylic acid (Trolox) (Cas No. 53188-7-1, Steinheim, Germany); Gallic acid (Cas No. 149-91-7, Steinheim, Germany); sodium carbonate (Na_2_CO_3_) (Cas No. 497-19-8, Germany, Merck); potassium persulfate (K_2_S_2_O_8_) (Cas No. 7727-21-1 Germany, Merck); sulfuric acid (H_2_SO_4_) (Cas No. 7664-93-9, Germany, Merck); Iron (III) chloride (FeCl_3_) (Cas No. 7705-08-0, Steinheim, Germany); were provided for this study with analytical grade.

### Extraction processes

#### Ultrasound-assisted enzymatic extraction processes

For the extraction of carotenoids, UAEE was performed with a laboratory scale ultrasonic bath (365 × 278 × 264 mm, W × D × H, Elmasonic S100H, 37 kHz, Singen, Germany). The tank of device (281 × 222 × 149 mm, WxDxH) is made of cavitation-resistant stainless steel which has filling volume is 9.50 l. Total power consumption of the device is 550W and maximum ultrasonic peak performance is 600 W. The temperature of bath can adjust by rotary switch from 30 to 80 °C and the change of temperature was controlled continuously with water-resistant digital thermometer with probe on cable during analysis. Sample (1–3 g) was mixed with 7 ml of extraction solvent (pectinase solution, 0–10%) and homogenized with ultra-turrax (IKA T18, Staufen, Germany) at third level speed (approximate 21.000 RPM/min) for 3 min. It was subjected to UAE using with determined temperature values and times by RSM. Independent process variables were selected as sample quantity, (1–3 g, *X*_*1*_), enzyme ratio (0–10%, *X*_*2*_), extraction temperature (20–60 °C, *X*_*3*_) and extraction time (10–60 min, *X*_*4*_) in the study. The experimental design of UAEE is shown in Table 1. At the end of extraction, all samples were kept in a water bath at 90 °C for 3 min for enzyme inactivation, and then cooled approximately 25 °C. Finally, samples were centrifuged (Universal 320 R, Tuttlingen, Germany) at 1968 × *g* for 10 min. After centrifugation, samples were filtered through a roughing filter paper. Afterwards, the filtered samples were kept at − 80 temperature until the chemical analysis stage.

**Table 1:** The experimental design of carotenoid extracts

Independent variables					Responses	
Run	Sample quantity (g) X_1_	Enzyme (%) X_2_	Temperature (ºC) X_3_	Time (min) X_4_	Carotenoid content (mg/kg)	b*
1	2	5	60	60	0.091(± 0.05)	13.41(± 0.01)
2	2	10	60	35	0.082(± 0.00)	17.88(± 0.00)
3	2	0	40	10	0.047(± 0.02)	9.18(± 0.00)
4	2	10	20	35	0.099(± 0.06)	19.99(± 0.02)
5	3	5	40	60	0.071(± 0.04)	19.97(± 0.00)
6	1	5	40	60	0.126(± 0.01)	9.26(± 0.01)
7	3	5	20	35	0.085(± 0.01)	18.9(± 0.02)
8	3	5	60	35	0.052(± 0.03)	16.25(± 0.03)
9	2	10	40	60	0.098(± 0.01)	16.05(± 0.03)
10	2	5	40	35	0.102(± 0.08)	20.01(± 0.06)
11	1	10	40	35	0.131(± 0.01)	11.37(± 0.01)
12	2	5	60	10	0.085(± 0.02)	16.68(± 0.02)
13	1	5	40	10	0.113(± 0.11)	12.67(± 0.02)
14	3	10	40	35	0.093(± 0.08)	22.75(± 0.01)
15	2	5	20	10	0.102(± 0.12)	15.11(± 0.03)
16	2	0	40	60	0.054(± 0.09)	14.41(± 0.07)
17	3	5	40	10	0.073(± 0.04)	15.89(± 0.02)
18	2	5	40	35	0.099(± 0.15)	20.14(± 0.01)
19	2	10	40	10	0.101(± 0.03)	20.51(± 0.01)
20	2	0	20	35	0.074(± 0.00)	12.88(± 0.05)
21	3	0	40	35	0.044(± 0.02)	12.03(± 0.07)
22	1	5	20	35	0.118(± 0.05)	10.97(± 0.00)
23	1	5	60	35	0.110(± 0.12)	11.78(± 0.02)
24	2	5	40	35	0.095(± 0.13)	19.52(± 0.02)
25	2	5	40	10	0.089(± 0.05)	19.59(± 0.06)
26	2	5	20	60	0.099(± 0.01)	17.95(± 0.01)
27	1	0	40	35	0.074(± 0.07)	9.93(± 0.02)
28	2	5	40	35	0.091(± 0.10)	19.49(± 0.08)
29	2	0	60	35	0.041(± 0.06)	12.42(± 0.04)

### Enzymatic extraction process

In the study, UAEE process was applied by selecting the optimum point determined according to the Design Expert 13 program. To determine the effectiveness of the ultrasonic process at the optimum point the extraction was applied by eliminating the ultrasound application under the same processes and conditions. For this reason, enzymatic extraction (EE) was carried out at the optimum point of UAEE. In the EE, Pectinex Ultra Color (Novozymes-Denmark) enzyme preparate was used for extraction of carotenoid from PP. Pomace was mixed with 7 ml of extraction solvent (pectinase solution, 8.5%) and homogenized with ultra-turrax at third level speed (approximate 21.000 RPM/min) for 3 min. This mixture was treated with heat at selected temperature within selected time according to optimum point of samples. After that for enzyme inactivation, all samples were kept in a water bath at 90 °C for 3 min and then cooled approximately 25 °C, then samples were centrifuged at 1968 × *g* for 10 min. And finally, the samples, which were passed through the roughing filter paper, were kept at − 80 temperature until the chemical analysis stage.

### Analytical determinations

#### Physicochemical analyses

The pH, titratable acidity (TA) and total soluble solids (TSS) of carotenoid extracts were determined according to the AOAC (AOAC 1995). pH values of the carotenoid extracts were analyzed by using a pH meter (WTW Inolab, Germany). TA was determined by titrimetric method and expressed as the percentage of malic acid (%). TSS of extracts were recorded with a digital refractometer (RFM 330; Bellingham Stanley Limited, Atago-Palette, PR-101, Tokyo, Japan) at 20 °C. The results were expressed in ºBrix.

### Color

Color measurements were analyzed in a Minolta colorimeter (Minolta, CR-300, Osaka, Japan). The color parameters L* (darkness, lightness), a* (redness, greenness), b* (blueness, yellowness) were determined according to CIELAB color co-ordinates. Total color differences (ΔE) and chroma (ΔC) values were determined according to control group, untreated PP. ΔC and ΔE were determined according to Eqs. (1) and (2).

1 $${\Delta C} = {\text{[(a}} - {\text{a}}_{{{\text{ref}}}} {)}^{{2}} {\text{ + (b}} - {\text{b}}_{{{\text{ref}}}} {)}^{{2}} {]}^{{1/2}}$$

2 $${\Delta E} = {\text{[(L}} - {\text{L}}_{{{\text{ref}}}} {)}^{{2}} {\text{ + (a}} - {\text{a}}_{{{\text{ref}}}} {)}^{{2}} {\text{ + (b}} - {\text{b}}_{{{\text{ref}}}} {)}^{{2}} {] }^{{1/2}}$$

### Total phenolic compounds

Carotenoid extracts for total phenolic compounds analysis were conducted by the method of Singleton and Rossi (1965). Firstly, 500 µL of extract was blending with 2 mL of Folin–Ciocalteau reagent (10% v/v). This mixture was blended with 1 mL of Na_2_CO_3_ solution (7% v/v) and kept in a place without light at room temperature for 30 min. At the end of this period, absorbance was taken at 760 nm wavelength (PG Instruments, Leicestershire, United Kingdom). Standard curves were created from the concentrations of gallic acid using standard response to the absorbance values read at 760 nm. The concentration value of samples corresponding to the absorbance from the standard calibration chart was calculated taking dilutions into account and defined as mg gallic acid/L. TPC results were determined according to Eq. (3) obtained from calibration chart (*R*^2^ value: 0.9992).

3 $${\text{y}} = {0}{\text{.0121x}} + {0}{\text{.1245}}$$

### Antioxidant activity

The antioxidant activity of the samples was determined by ABTS and ferric reducing antioxidant power (FRAP) methods. For ABTS method, Trolox was used as standard. Stock solutions of 7.0 mM ABTS and 2.45 mM potassium persulfate (K_2_S_2_O_8_) were prepared. Working solution was obtained by mixing the prepared 7 mM ABTS stock solution and 2.45 mM K_2_S_2_O_8_ solution in equal proportions and this working solution incubated at room temperature for 17 h in the dark. And then, 1 mL of this solution was diluted by mixing with 50 mL 20 mM sodium acetate (pH 4.5) to obtain 0.700 ± 0.05 absorbance value at 734 nm. After that extracts (10 μL) were allowed to react with 2990 μL of adjusted solution (absorbance value of 0.700 ± 0.05) for 30 min at room temperature in a dark condition. The absorbance of extracts was read at 734 nm using by spectrophotometer. The obtained data were calculated on a standard curve and expressed as mg Trolox/L (Pająk et al. 2019).

The analysis of the FRAP was conducted according to Benzie and Strain (1996). Briefly, working solution was prepared by stirring 0.3 M sodium acetate (pH 3.6), 10 mM TPTZ and 20 mM Iron (III) chloride in a 10:1:1 ratio. After that, 100 µL extract was allowed to react with 2900 μL of working solution for 30 min in a dark place for 30 min at room temperature. The absorbance was taken at 593 nm. The results obtained with using a standard curve were expressed as µmol Trolox/L.

### Total carotenoid content

To determine the total carotenoid values of the peach extracts, the spectrophotometric method which developed by Lee and Castle (2001) was used. Firstly, 15 mL extract was blending with 30 mL extraction solvent (hexane/acetone/ethanol; 50/25/25 v/v) and homogenized by using ultra-turrax for 30 s. The mixture was centrifuged at 1968 × *g* (EBA 21, Tuttlingen, Germany) for 10 min at 5 °C. And then samples were measured at 450 nm wavelength. The carotenoid content was calculated in ppm β-carotene according to the formula Eq. 4, taking into account the molar absorption coefficient (E^1%^; E_1cm_ = 2505).

4 $${\text{c}} = \left( {{\raise0.7ex\hbox{${\text{a}}$} \!\mathord{\left/ {\vphantom {{\text{a}} {\text{E}}}}\right.\kern-0pt} \!\lower0.7ex\hbox{${\text{E}}$}} \times {\text{b}}} \right) \times {1000}$$

c: unit concentration (w/v).

a: absorbance value.

E: molar absorption coefficient, 2505.

b: unit optical path length, 1 cm.

### Optimization and statistical analysis

The RSM was practiced for the optimization of carotenoid extraction process using a desirability function approach for maximum total carotenoid content and b* (yellowness) color value of peach extracts. Box-Behnken trial pattern with four factors (*X*_1_, sample quantity; *X*_2_, enzyme ratio; *X*_3_, extraction temperature; *X*_4_, extraction time) was applied in at three levels to determine the optimum UAEE conditions. 29 different runs were conducted in triplicate at the center point within RSM design program. All of the results of these runs were evaluated in terms of regression coefficient, regression equations and statistical analysis of variance by using the software Design-expert 13.0 (Stat-Ease Inc., USA) for statistical analysis of variance, regression coefficients and regression equations. Each experiment was performed in triplicate.

The significant difference between the means of obtained analysis results was examined by ANOVA variance analysis and Duncan Tests. All results were expressed as the mean ± standard deviation of three separate experiments. The results were evaluated with the SPSS statistical package program (SPSS 17.0 for Windows Version, SPSS Inc., Chicago, USA). The fit of the polynomial model equation was stated by the coefficient of determination* R*^2^ and its statistical significance was checked by an *F*-test.

## Results and discussion

### Statistical analysis and the model fitting

**Fig. 1.**
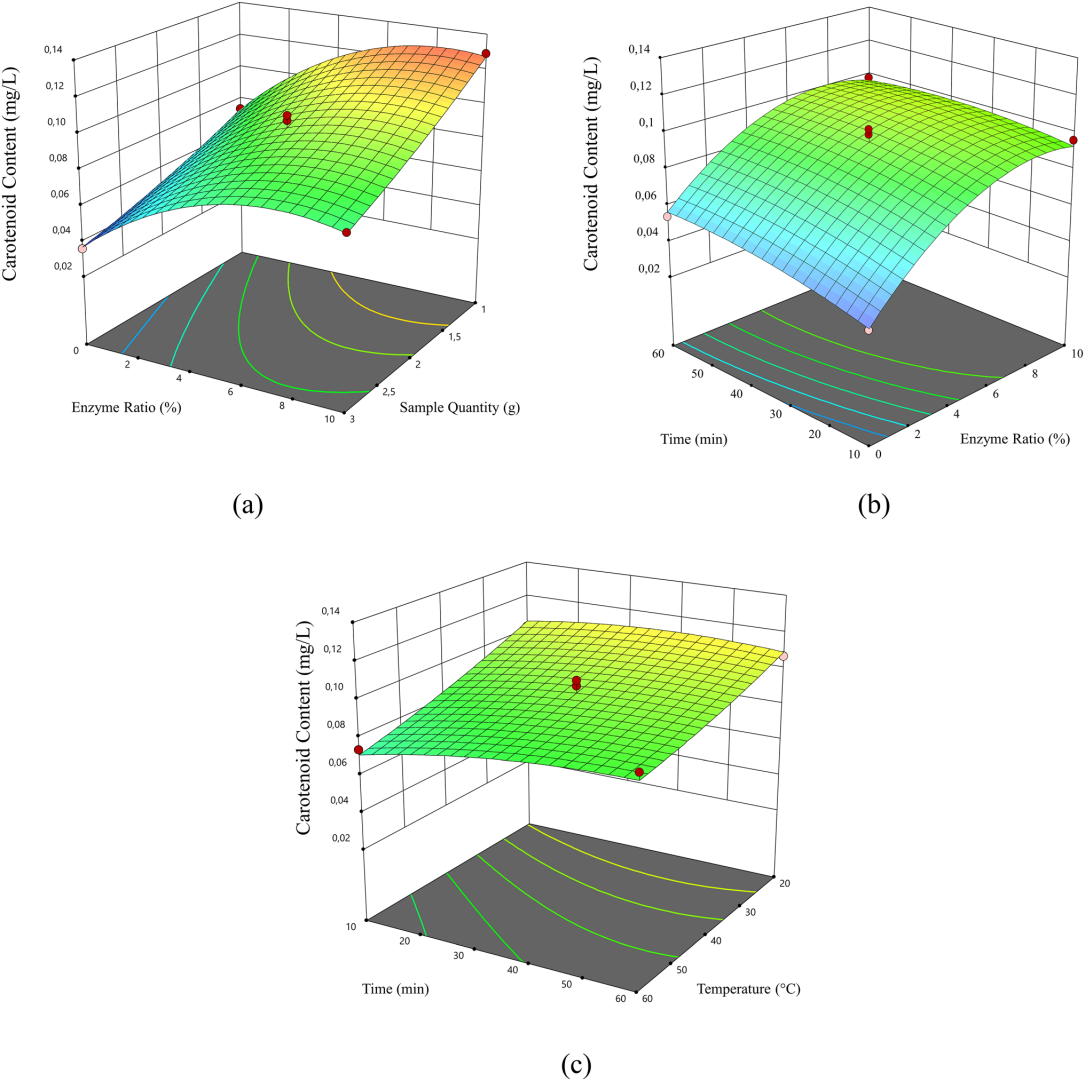
3D response surface graphics of carotenoid content in the optimization phase. **a** Enzyme ratio-sample quantity, **b** extraction time–enzyme ratio, **c** extraction time–extraction temperature

**Fig. 2.**
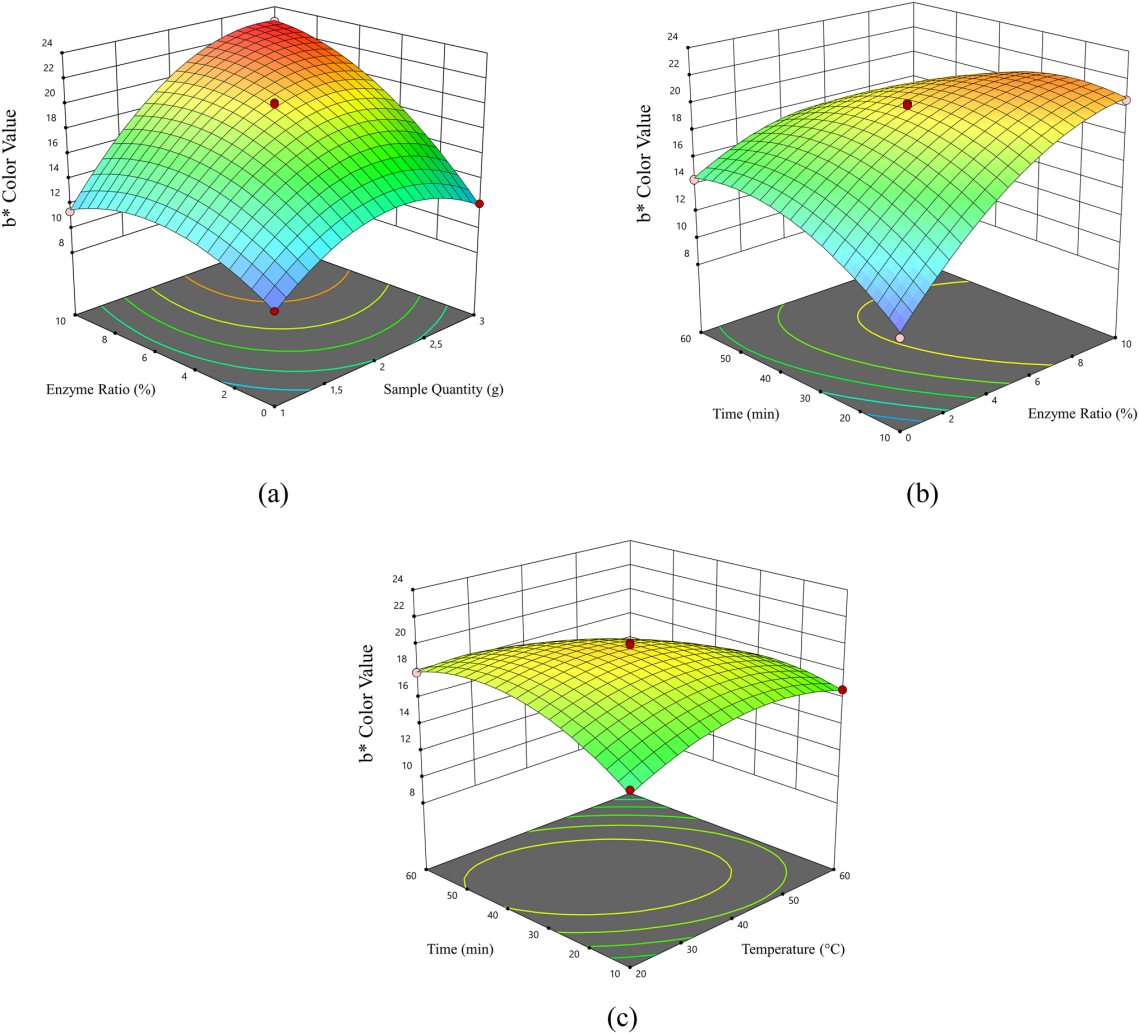
3D response surface graphics of b* color value in the optimization phase. **a** Enzyme ratio-sample quantity, **b** extraction time–enzyme ratio, **c** extraction time–extraction temperature

The data obtained for the carotenoid content and b* color value analyzes performed within the scope of the experimental design are given in Table 1. The response surface graphs indicating the effects of independent variables to carotenoid content and b* color value are shown in Figs. 1 and 2, respectively. When extraction temperature and time were kept constant at a certain temperature and time, the carotenoid content was increased up to certain point as the enzyme ratio increased. On the other hand, the carotenoid content was decreased as the sample quantity increased. The correlation can also be seen in Fig. 1a where the response surface graphs of carotenoid content. Studies on the increase of extraction efficiency with the use of enzymes also support the results obtained (Nath et al. 2016). In another similar study, it was determined that the use of commercial proteases and carbohydrase increased the biomass yield by 20–45% compared to solvent extraction. Enzymatic reaction contributes to disruption of plant cell wall (Kulshreshtha et al. 2015). Pectin esterase, pectin lyase and polygalacturonase due to their pectin-degrading activity increase breakdown of the pectic substance. This results in free run of juice. For this reason, the carotenoid content gradually increased depending on ultrasound process time, especially at higher values above 30 min. This situation can be seen in Fig. 1b and c. The carotenoid content and b* color value reached a maximum at 40 ℃. But these values were declined with increasing temperature (Figs. 1c and 2c). This might be because, rise in temperature result in the intensification of molecular motion, by this way increasing the solubility rate of compounds in pomace. But at higher temperatures degradation of carotenoids happens. Similar experimental results were obtained by other authors in the case of carotenoids from Sesbania grandiflora leaves, oil palm fronds and rapeseed using different temperature treatments (Gunathilaka et al. 2017). It was also determined that the b* color value was increased up to a certain value depending on the raising in the sample quantity and enzyme ratio (Fig. 2a). When the impact of temperature was examined, it was concluded that the ultrasound combined with warm heat treatment, neither too hot nor too low temperature degrees, have significant impact on carotene extraction. This phenomenon accelerates extraction of cellular contents because of cavitation resulting in degradation of cell walls (Wang et al. 2021; Xue et al. 2021). For this reason, in the extraction process, the process conditions obtaining the highest total carotenoid content and b* color value of each group were optimized using desirability function. In this sense, three optimum points were selected providing the 85–100% condition by desirability and among these points reached the highest values when the sample quantity was 1.00 g, enzyme ratio was 8.50%, the extraction temperature was 45.18 ºC, and the extraction time 50.36 min. The carotenoid extract was reproduced. The maximum carotenoid content and b* color value were calculated, as shown in Table 2, and the optimum production parameters were determined.

**Table 2:** ANOVA table and statistical parameters of carotenoid content and b* color value of extracts

	df^a^	Coefficient		Sum of squares		*p*-value	
		Carotenoid	Color b*	Carotenoid	Color b*	Carotenoid	Color b*
Model	14	0.0989	− 18.02	0.0181	433.94	< 0.0001	< 0.0001
x_1_	1	− 0.0147	+ 13.42	0.0059	132.07	< 0.0001	< 0.0001
x_2_	1	+ 0.0145	+ 1.4943	0.0063	118.44	< 0.0001	< 0.0001
x_3_	1	− 0.0011	+ 0.5381	0.0024	4.54	< 0.0001	< 0.0001
x_4_	1	+ 0.0010	+ 0.3151	0.0003	0.0850	0.0010	0.4139
x_1_ x_2_	1	− 0.0007	+ 0.4640	0.0001	21.53	0.0901	< 0.0001
x_1_ x_3_	1	− 0.0001	− 0.0432	0.0000	2.99	0.2449	0.0002
x_1_ x_4_	1	− 0.0002	+ 0.0749	0.0002	14.03	0.0089	< 0.0001
x_2_ x_3_	1	+ 2.5 × 10^–4^	− 0.0041	2.5 × 10^–5^	0.6806	0.9051	0.0319
x_2_ x_4_	1	− 0.0000	− 0.0193	0.0000	23.47	0.1673	< 0.0001
x_3_ x_4_	1	+ 6.0 × 10^–3^	− 0.0030	0.0000	9.33	0.1673	< 0.0001
x_1_^2^	1	+ 0.0025	− 3.3300	0.0000	71.95	0.1408	< 0.0001
x_2_^2^	1	− 0.0007	− 0.0950	0.0024	36.64	< 0.0001	< 0.0001
x_3_^2^	1	+ 5.37 × 10^–4^	− 0.0044	0.0000	20.42	0.2050	< 0.0001
x_4_^2^	1	− 6.56 × 10^–3^	− 0.0034	0.0001	30.42	0.0238	< 0.0001
Residual	14			0.0002	1.68		
Lack of Fit	10			0.0001	1.31	0.8826	0.3887
Pure error	4			0.0001	0.3658		
Cor total	28			0.0183	435.61		
*R*^2^		0.9870	0.9961				
Adj *R*^2^		0.9740	0.9923				
Pred *R*^2^		0.9520	0.9813				
PRESS		1.1*10^–3^	8.13				
CV		4.71	2.20				
Adequate Precision		32.5878	54.9117				

*df* degrees of freedom, *p* > 0.05 indicate no significant differences, *p* < 0.05 indicate significant differences, X_1_ sample quantity (g), X_2_ enzyme (%), X_3_ extraction temperature (°C), X_4_ extraction time (min)

The results of the variance analysis of the RSM are given in Table 2 for carotenoid content and b* color value of extracts. The ANOVA results for response surface quadratic model and multiple regression analysis are evaluated using the corresponding *p-*values. According to Table 2, the model created in experiment was significant at the ratio of *p* < 0.01 which demonstrates that the model is statistically significant. The lack of fit was not significant (*p* > 0.05) at 95% confidence level, indicating that the model was suitable. Additively, *R*^2^, Adj-*R*^2^, Pre-*R*^2^ and the coefficient of variation (CV) are assessed to control model adequacy. The coefficient of determination (*R*^2^) of carotenoid and b*color values were 0.9870 and 0.9961, respectively demonstrated that the model explained about 99.0% of the variances of response values. Also, Pre-*R*^2^ and Adj-*R*^2^ values determined for carotenoid and b* color parameter are close and compliance with each other. This indicates that there is a high degree of correlation between the real and predicted data in the created regression model (Wu et al. 2014). The CV value, which expresses the distribution of experimental points according to the model propositions, was found to be 4.71 and 2.20% (< 5.00%) for carotenoid content and b* color value. This shows that the model is reproducible. Adequate precision expressing the signal-to-noise ratio were determined as 32.58 and 54.91 for carotenoid content and b* value, respectively, in this study. These values indicates that the model is significant for the present UAEE process (Jin and Ning 2013). When all these situations are evaluated, the model created can be used for the prediction and optimization of carotenoid extraction yield with maximum b* color value under different independent factors during UAEE. According to model for carotenoid content, it can be seen clearly that the independent variables (*X1*, sample quantity, *X*_*2*_ enzyme ratio, *X*_*3*_ extraction time and *X*_*4*_ extraction temperature), the interaction terms (*X*_*1*_*X*_*4*_), and quadratic terms (*X*_*2*_^*2*^*)* affected the carotenoid content (*p* < 0.05) based on the analysis of validity. The interaction term of sample quantity and extraction time (*p* < 0.05) have significant effect on the carotene content of extracts. In quadratic interactions of the independent variables, enzyme content and extraction time values are significant conversely sample quantity and extraction temperature seems not to be significant (*p* > 0.05).

According to Table 2 for color b*, the model established in this experiment was extremely significant (*p* < 0.01), and the lack of fit was not significant (*p* > 0.05) and *R*^2^value indicate that the model expressed about 99.0% of the response changes. It can be showed in Table 2 that the sample quantity, enzyme ratio, extraction time and extraction temperature affected the b* color values (*p* < 0.05). The linear and quadratic interaction terms of independent variables except extraction time all affected b* color value of extracts (*p* < 0.05).

A second-order polynomial model was generated for carotenoid content and b* color values were chosen in response. The model equations of UAEE in terms of coded factors is given in Eqs. (5) and (6).

5 $${\text{Carotenoid content }}\left( {\text{mg/kg}} \right){ = 0}{\text{.0989 }} - { 0}{\text{.0147X}}_{{1}} { + 0}{\text{.0145X}}_{{2}} { } - {0}{\text{.0011X}}_{{3}} { } - { 0}{\text{.0010X}}_{{4}} { } - { 0}{\text{.0025X}}_{{1}}^{{2}} - { 0}{\text{.0007X}}_{{2}}^{{2}} { } - { 6}{\text{.56x10}}^{{ - 3}} {\text{X}}_{{4}}^{{2}} { } - { 0}{\text{.0002X}}_{{1}} {\text{X}}_{{4}} { }$$

6 $${\text{Color b*}} = - {18}{\text{.02 + 13}}{\text{.42X}}_{{1}} { + 1}{\text{.4943X}}_{{2}} { + 0}{\text{.5381X}}_{{3}} { + 0}{\text{.3151X}}_{{4}} { } - { 3}{\text{.33X}}_{{1}}^{{2}} - { 0}{\text{.0950X}}_{{2}}^{{2}} { } - {0}{\text{.0044X}}_{{3}}^{{2}} { } - { 0}{\text{.0034X}}_{{4}}^{{2}} { + 0}{\text{.4640X}}_{{1}} {\text{X}}_{{2}} { } - { 0}{\text{.0432X}}_{{1}} {\text{X}}_{{3}} { + 0}{\text{.0749X}}_{{1}} {\text{X}}_{{4}} { } - {0}{\text{.0041X}}_{{2}} {\text{X}}_{{3}} { } - {0}{\text{.0193X}}_{{2}} {\text{X}}_{{4}} { } - { 0}{\text{.0030X}}_{{3}} {\text{X}}_{{4}} { }$$

(X_1_ sample quantity (g); X_2_ enzyme (%); X_3_ extraction temperature (ºC); X_4_ extraction time (min)).

### Physicochemical, color and antioxidant analyses of carotenoid extracts

#### Physicochemical analyses

Analysis results of physicochemical, color and antioxidant activity are shown in Table 3. When pH values of samples examined, the pH of PP as control group was 4.06 ± 0.02 and 3.86 for the extract samples obtained by UAEE and EE methods. When the obtained data are evaluated statistically; while the difference between the pH value of the control group and extract samples obtained by UAEE and EE was significant, no difference was detected when UAEE and EE methods were evaluated within themselves (*p* > 0.5). Küçüker et al (2015) also reported similar pattern where the pH value was 3.96–4.10 depending on type of peach. While there was no statistical difference in the TA values between extracts. TA value of extract obtained by UAEE was lower than EE. It is thought that the reason for this situation is due to the breakdown of organic acids in the composition by the ultrasonic extraction method. Considering the similar studies on this subject, pH and TA values obtained within the scope of present study were within the expected value ranges (Bayazıt et al. 2012). When evaluated the results of total soluble solids of extracts, they were decreased within applied both extraction processes. The brix values of the extracts obtained are lower, since no concentration process is applied.

**Table 3:** Analy**s**i**s** results of enzymatic and ultrasonic-assisted enzymatic extraction of extracts

		Carotenoid extracts	
Analyses	Peach pomace (Control)	Enzymatic extraction	Ultrasonic-assisted enzymatic extraction
*Physicochemical*			
pH	4.06 (± 0.02)^a^	3.86 (± 0.00)^b^	3.86 (± 0.01)^b^
TA	1.07 (± 0.07)^a^	0.99 (± 0.00)^b^	0.86 (± 0.06)^b^
TSS	11.00 (± 0.10)^a^	6.85 (± 0.23)^c^	4.13 (± 0.06)^b^
*Color*			
L*	49.28 (± 3.27)^a^	45.34 (± 0.03)^b^	45.02 (± 0.03)^b^
a*	8.92 (± 1.73)^a^	− 0.13 (± 0.02)^b^	− 0.12 (± 0.03)^b^
b*	37.37 (± 4.07)^a^	4.93 (± 0.26)^b^	5.04 (± 0.15)^b^
Chroma value (∆C)	–	33.57(± 0.03)^a^	33.68(± 0.14)^a^
Color difference (∆E)	–	33.80(± 0.03)^a^	33.95(± 0.13)^a^
TPC	2114.60 (± 12.02)^a^	698.51 (± 6.09)^c^	761.10 (± 8.12)^b^
*Antioxidant activity*			
ABTS	7435.90 (± 21.03)^a^	1918.97 (± 10.11)^b^	1933.33 (± 11.06)^b^
FRAP	336.19 (± 9.08)^a^	49.44 (± 0.79)^b^	52.66 (± 1.65)^b^

*TA* titratable acidity (malic acid %), *TSS* total soluble solids (brix %), *TPC* total phenolic content (mg gallic acid/L dry sample), *ABTS* antioxidant activity (mg trolox/L), *FRAP* antioxidant activity (µmol trolox/L) β-carotene, *lycopene* lutein carotenoid distribution (µg/100 g)

^a^^−c^Means with uncommon superscripts within a line are significantly different (*p* < 0.05)

#### Color

According to Table 3, the values obtained for the L* color parameter of extracts were determined 45.02 ± 0.03 and 45.34 ± 0.03 by UAEE and EE method, respectively and L* color value of PP, which was not applied any extraction process, was specified 49.28 ± 3.27. Both extraction processes reduced L* color. It is thought that the reason for this situation is that the pectinase enzyme breaks down the cell walls of the peach pomace, allowing more carotenoids to be taken from it. Similar study on the subject proves that the use of pectinase in extraction processes can decrease L* color value of extracts (Chen et al. 2012). And also, it was determined that results of this study are complied with findings of Shahram and Dinani (2019). When considering similar studies, it is thought that reduction of L* color value of extracts with extension of applied ultrasound time can be based upon to the oxidation of the compounds in extracts. This is supposed to mean the oxidation of the antioxidant compounds in the carotenoid extracts induces reducing the L* color values of them. In color analyses a* parameter is relative to the green–red colors, with negative values toward green and positive values toward red. In this sense, it was determined that a* values of both extracts in present study were negative and against this it was positive in PP. The obtained b* color value of extracts was 7.36 ± 0.26 and 8.99 ± 0.15 from EE and UAEE, respectively. The highest b* color parameter of the extracts was observed with using UAEE method. These results are in line with the findings of similar studies on the subject (Shahram and Dinani 2019). Higher ultrasound frequency causes increasing extracted carotenoid content and more yellow pigment to pass into the extract, thus increasing the b* color value. When ∆C and ∆E values of extracts were assessed, there was no statistically difference between two extraction methods. But in despite of being a slight difference, the values are higher in the extract obtained by the UAEE method. It was determined that the samples obtained by ultrasonic method contained higher amount of color pigment. This could be based upon the cavitation event caused by ultrasound. The shear stress due to cavitation accelerates the transition of bioactive compounds like carotenoids to solvent (Bansode and Rathod 2017).

#### Total phenolic compounds

When obtained TPC value of carotenoid extracts were compared, the highest value (761.10 ± 8.12 mg gallic acid/L) was reached by UAEE method (Table 3). TPC results were consistent with previous studies and it has been determined that ultrasound has a positive contribution on phenolic content (Teh and Birch 2014). González-Centeno et al (2014) reported that the maximum phenolic extraction yield was performed at low ultrasound frequencies with increasing extraction time. It is thought that the reason for this event is that ultrasonic irradiation accelerates enzymatic pretreatment. With the ultrasound process applied to the extracts, the extraction yield increases with the cavitation that occurs as result of the ultrasonic waves spreading to the solvent medium. Also, ultrasonic treatment under optimum conditions can caused to increase enzyme activity. This phenomenon is by reason of favorable conformational changes and structural integrity which improve the extraction process of biomolecules and facilitate solvent access to the cell content intensifying the mass transfer rate (Nadar and Rathod 2017; Savic Gajic et al. 2019; Vázquez-Espinosa et al. 2019). This is in line with the studies of reported for UAE of phenolics from mango peels (Martínez-Ramos et al. 2020), makiang seeds (Sirichan et al. 2022), and kiwi fruit (Wang et al. 2019).

#### Antioxidant activity

Carotenoids, which are expressed as protective agents against cardiovascular and neurological diseases as well as various cancer diseases, act as protective role in various reactive oxygen species (ROS). Antioxidant activities of carotenoids are varied widely because of having many mechanisms to inactivate the effects of ROS (Mussagy et al. 2019). When antioxidant capacity analyses (ABTS and FRAP) results of extracts are examined, the highest values (1933.33 ± 11.06 mg Trolox /L and 52.66 ± 1.65 µmol Trolox /L) were obtained with UAEE method. Similarly, the study found that the total antioxidant capacity of kiwi fruits (53.57–121.88 μmol/100 mL) significantly increased with ultrasound treatments compared to the untreated samples (Wang et al. 2019). In another study conducted with grape pomace, it was determined that there was a linear increase in antioxidant capacity values with the increase of ultrasound frequency and extraction time, and the highest value was recorded as 43.66 mg Trolox/100 g pomace (González-Centeno et al. 2014). It is thought that this situation might be occurred due to the deterioration of the cell walls of fruit tissues with the effect of ultrasonication, which leads to more release of antioxidants (e.g., phenolics) into the samples. In addition, inactivation of some oxidation related enzymes like polyphenol oxidases resulted from cavitation formed during ultrasound, which would also result in an increase of total antioxidant capacity in fruit juice (Rakshit and Srivastav 2023). No study has previously extracted carotenoids from PP by UAEE method. However, in a study on carotenoid extraction from orange processing wastes, it was found that the antioxidant activity values of the extracts were similar and the highest value was obtained with pectinase concentration of 5% (w/w) and ultrasonic time of 10 min (Shahram and Dinani 2019). In another study conducted by Garcia-Castello et al (2015), it was determined that the total phenolics (%50) and antioxidant activity values of bioactive compounds obtained by UAE from grapefruit wastes were higher than other extraction methods. Thus, in the study, it was defined that the use of ultrasound provides higher recovery with lower temperature conditions and shorter processing time.

## Conclusion

In this study, the optimum conditions for extraction of carotenoid pigment from PP were investigated by comparing the ultrasonic method and the conventional method. In this sense, firstly, the effects of four parameters of sample quantity (1–3 g), enzyme ratio (0–10%), extraction temperature (20–60 °C) and extraction time (10–60 min) on total carotenoid content and b* color parameter of the extracts were evaluated by using RSM. The optimal experimental extraction yield was obtained when the sample quantity was 1.0 g, enzyme ratio was 8.50%, extraction temperature was 45.18 °C, and extraction time was 50.36 min. When the results of physicochemical, color, antioxidant activity analysis of carotenoid extracts were examined, it was determined that the UAEE was more effective than the enzymatic extraction. The highest TPC, ABTS and FRAP values were obtained by UAEE method. In this sense, this study could be used as a reference for carotenoid extraction from PP offering an efficient and environmentally friendly extraction procedure for food industry. Especially, carotenoid extracts from PP within the scope of study has a potential application as natural antioxidant and coloring agent for food formulations compatible with their acidic structure and it will contribute to the product protection and minimization of color losses. For this reason, it is necessary to carry out comprehensive studies on the microbial characteristic, thermal stability, light resistance, changes due to storage conditions and sensory evaluations of extracts.

## Data Availability

Data sharing is not applicable to this article as no datasetswere generated or analyzed during the current study.
